# Identifying key genes in CD4^+^ T cells of systemic lupus erythematosus by integrated bioinformatics analysis

**DOI:** 10.3389/fgene.2022.941221

**Published:** 2022-08-15

**Authors:** Zutong Li, Zhilong Wang, Tian Sun, Shanshan Liu, Shuai Ding, Lingyun Sun

**Affiliations:** ^1^ Department of Rheumatology and Immunology, The Affiliated Drum Tower Hospital of Nanjing University Medical School, Nanjing, China; ^2^ Department of Reproductive Medicine Center, The Affiliated Drum Tower Hospital of Nanjing University Medical School, Nanjing, China

**Keywords:** systemic lupus erythematosus, CD4^+^ T cells, disease activity, weighted gene co-expression network analysis, biomarker, type I interferon, immune response

## Abstract

Systemic lupus erythematosus (SLE) is a chronic autoimmune disease characterized by excessive activation of T and B lymphocytes and breakdown of immune tolerance to autoantigens. Despite several mechanisms including the genetic alterations and inflammatory responses have been reported, the overall signature genes in CD4^+^ T cells and how they affect the pathological process of SLE remain to be elucidated. This study aimed to identify the crucial genes, potential biological processes and pathways underlying SLE pathogenesis by integrated bioinformatics. The gene expression profiles of isolated peripheral CD4^+^ T cells from SLE patients with different disease activity and healthy controls (GSE97263) were analyzed, and 14 co-expression modules were identified using weighted gene co-expression network analysis (WGCNA). Some of these modules showed significantly positive or negative correlations with SLE disease activity, and primarily enriched in the regulation of type I interferon and immune responses. Next, combining time course sequencing (TCseq) with differentially expressed gene (DEG) analysis, crucial genes in lupus CD4^+^ T cells were revealed, including some interferon signature genes (ISGs). Among these genes, we identified 4 upregulated genes (*PLSCR1*, *IFI35, BATF2* and *CLDN5*) and 2 downregulated genes (*GDF7* and *DERL3*) as newfound key genes. The elevated genes showed close relationship with the SLE disease activity. In general, our study identified 6 novel biomarkers in CD4^+^ T cells that might contribute to the diagnosis and treatment of SLE.

## Introduction

Systemic lupus erythematosus (SLE) is a chronic autoimmune disease which affects diffuse connective tissues and organs, including skin, joints and kidneys. SLE is characterized by excessive activation of T and B lymphocytes and breach of immune tolerance to autoantigens, which trigger the production of autoantibodies and lead to the immune-complex related inflammation in multiple organs and tissues (Tsokos, 2011). Genetic factors and environmental triggers are believed to play important roles in the pathogenesis and progression of SLE (Goulielmos et al., 2018; Dorner and Furie, 2019). Nevertheless, the pathogenic mechanisms of SLE have not been fully understood yet. Thus, the time-course of disease flares, remission and progression is unpredictable (Obermoser and Pascual, 2010). Besides, SLE is also a highly heterogeneous disease in terms of diverse clinical manifestations and severity, which presents a challenge to the clinicians and researchers. It would therefore be of great value to explore the molecular signatures underlying different clinical phenotypes, as it could aid in accurate diagnosis, disease activity assessment and clinical management of SLE.

The mutation and abnormal expression of many vital genes also confer a predisposition to SLE, indicating the value of diagnosis or prognosis (Teruel and Alarcon-Riquelme, 2016; Luo et al., 2020). However, peripheral blood mononuclear cells (PBMCs) consist of a mixture of lymphocytes and monocytes, and rarely show a good enough discrepancy on transcriptomic profiles. Among the major peripheral immune cells in lupus, the autoreactive and pro-inflammatory CD4^+^ T cells stimulate the differentiation, proliferation and maturation of B cells to enhance the production of autoantibodies, playing a key role in the pathogenesis and progression of SLE (Moulton and Tsokos, 2011; Zhao et al., 2018; Jang et al., 2021). The alterations in the signaling physiology and gene transcription lead to abnormalities in the phenotype of these cells (Mak and Kow, 2014; Yuan et al., 2022). Most of all, the peripheral blood cells of lupus patients demonstrated overexpression of the gene profiles induced by type I interferon (IFN), also known as interferon signature genes (ISGs) (Feng et al., 2015; Ronnblom, 2016; Postal et al., 2020). However, how these molecular signatures correlate with SLE activity awaits further characterization. The transcriptomic or translational profiles of lupus CD4^+^ T cells can lead to a better understanding of pathogenic mechanisms of SLE, and aid in potential therapeutic targets identification in an unbiased manner.

Weighted gene co-expression network analysis (WGCNA) is a well-known method of systems biology for exploring and identifying the potential functional pathways and biomarkers for diagnosis and prognosis of complex diseases at the level of the genome (Langfelder and Horvath, 2008). This powerful bioinformatic tool has been widely used in various diseases, including SLE and other autoimmune diseases (Yan et al., 2018; Sun et al., 2019). Using WGCNA, Liu *et al.* found overexpressed small RNAs encoded by human endogenous retrovirus K in PBMCs that might be involved in the immune regulation and progression of SLE (Liu et al., 2021). Similarly, *IFI27* may be closely related to pathogenesis of SLE (Zhao et al., 2021). In lupus nephritis (LN), the potential gene expression biomarkers for diagnosis and prognosis were developed by integrating multiple differentially expressed gene (DEG) identification methods (Yao et al., 2020; Chen et al., 2021; Shen et al., 2021). In this study, through integrated bioinformatics analysis of high-throughput sequencing data, we well-characterized the gene expression profiles in CD4^+^ T cells obtained from healthy controls (HC) and lupus patients, and identified crucial genes correlated with the severity of SLE. These findings could improve our understanding of the disease pathogenesis and provide new insights in identification of potential diagnostic and therapeutic molecular targets of SLE.

## Materials and methods

### Data acquisition and processing

We obtained the gene expression dataset GSE97263 with corresponding clinical information from the GEO database (https://www.ncbi.nlm.nih.gov/geo/query/acc.cgi?acc=GSE97263), which was performed with the platform of Illumina HiSeq 2500. This dataset contained isolated blood CD4^+^ T cells from 14 HC, 14 active and 16 inactive SLE patients (Buang et al., 2021). The Systemic Lupus Erythematosus Disease Activity Index (SLEDAI score) was used for clinical classification. Inactive SLE was defined as a SLEDAI <4 and active SLEDAI >6. Processing and analysis of these collected data were conducted with the R software. The Ensemble IDs were converted into gene symbols with the bitr function in clusterProfiler (Wu et al., 2021), and the genes with average raw reads value less than 1 were removed. After data processing, 16,623 genes were matched, and all these genes were used for the following WGCNA after normalized with log transformed (in detail, log(edgeR:cpm(counts+1)).

### WGCNA

A sample clustering tree map was first constructed to detect and eliminate outliers. Then, the “WGCNA” R package (Langfelder and Horvath, 2008) was used to construct the gene network with the dataset GSE97263. In detail, scale independence and mean connectivity were identified *via* the soft threshold power (*β* value) setting of 1–20. Meanwhile, soft threshold power was selected as the degree of scale independence reached 0.85. Based on the selected soft threshold, the adjacency matrix was converted to topological overlap matrix (TOM) to construct the network. Then, we performed module identification using cutreeDynamic function with minClusterSize = 100, and the gene dendrogram and module color were established using the degree of TOM-based dissimilarity(1-TOM). Next, MergeCutHeight function was used for cutting the dendrogram in the process of module merging with the a 0.25 MEDissThres value, and 14 modules were finally harvested.

### Identification of clinically significant modules

The Pearson correlation coefficient between the module eigengene (ME) and sample traits was calculated to find out the highly relevant module (hub module) associated with the development of SLE. Modules with top 2 corresponding correlation with *p* < 0.05 were identified. Then the correlationship between Module Membership (MM, the correlation of the module eigengene and the gene expression profile) and Gene Significance (GS, the correlation between the gene and the clinical phenotypes) in these modules was calculated and visualized with the “WGCNA” R package (Langfelder and Horvath, 2008). The significantly correlation GS and MM implied that hub genes of the modules tend to be highly correlated with clinical phenotypes.

### Functional enrichment analysis

In order to identify the function of the selected genes in the pathogenesis and development of SLE, Kyoto Encyclopedia of Genes and Genomes (KEGG) and Gene Ontology (GO) enrichment analysis of gene functions in hub modules were performed using the “clusterProfiler” R package (Wu et al., 2021) with the default parameters.

### Identification of DEGs in HC, inactive and active SLE

The “DESeq2” R package was used to identify DEGs among the three groups with the threshold of |log_2_FC| >1 and *p* < 0.05. Besides, the “TCseq” R package “timeclust” function was used to characterize the gene expression patterns in three groups following the manuscript (DOI: 10.18129/B9.bioc.TCseq). The clusters that showed positive or negative relations with SLE activity were selected.

### Identification of key genes

The Jvenn web tool was used to identify the overlapped intersection of the genes identified by WGCNA, time course sequencing (TCseq) and DEG analysis. These key genes were selected to perform principal component analysis (PCA) and heatmap analysis for validation.

### Identification of potential biomarkers of SLE

First, we analyzed the key genes identified above with the “clusterProfiler” R package (Wu et al., 2021), and identified the genes that participated in regulation of top GO terms related to the development of SLE. Then, we compared the gene expression between HC and SLE patients with different disease activity and identified the potential biomarkers of diagnostic and therapeutic value for SLE.

### Statistical analysis

All the statistical process and analysis in this manuscript were performed with R software, and *p* < 0.05 was considered as statistically significant.

## Results

### Gene expression profiles in HC, inactive and active SLE

**FIGURE 1:**
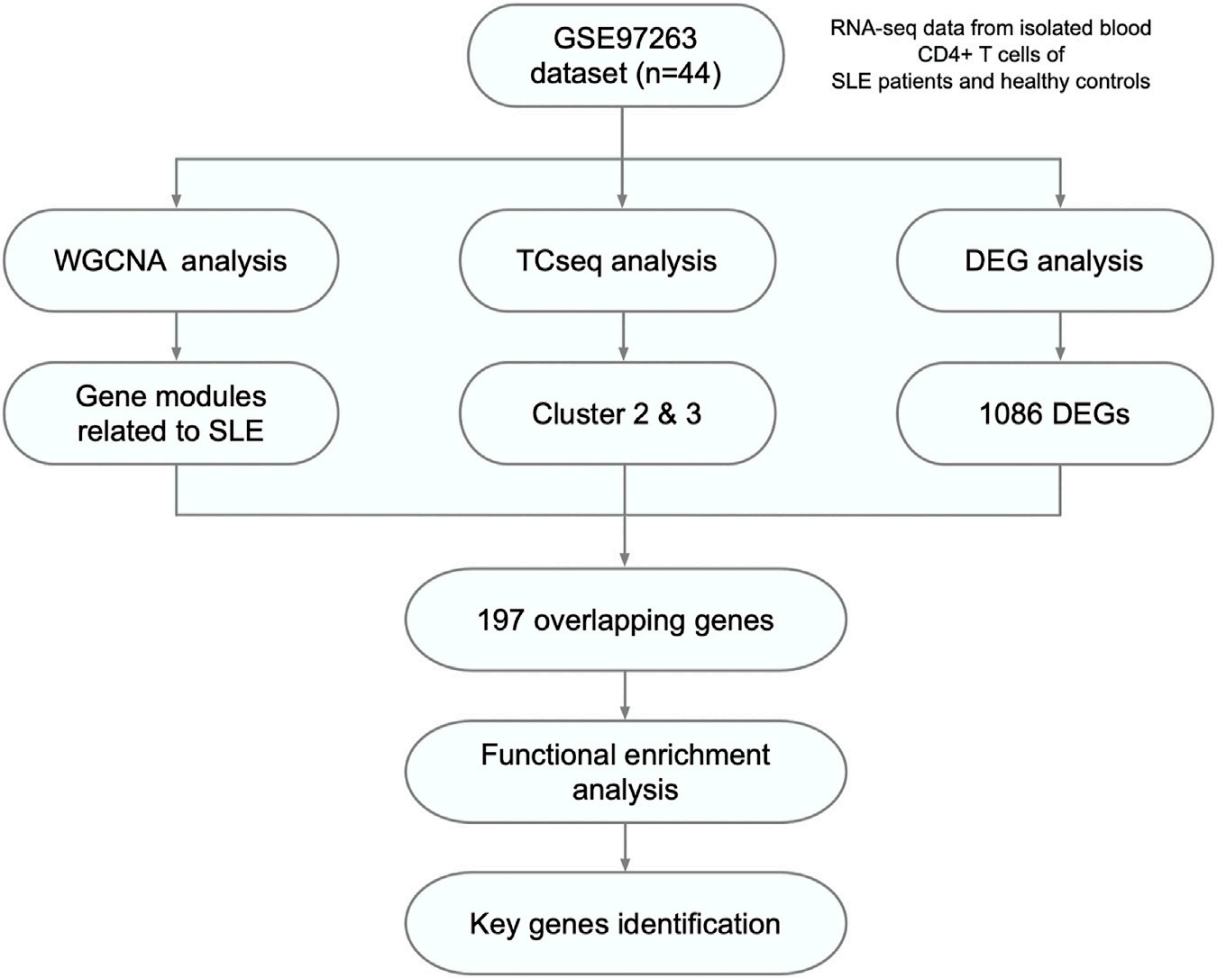
Study design and the workflow of this study.

**FIGURE 2:**
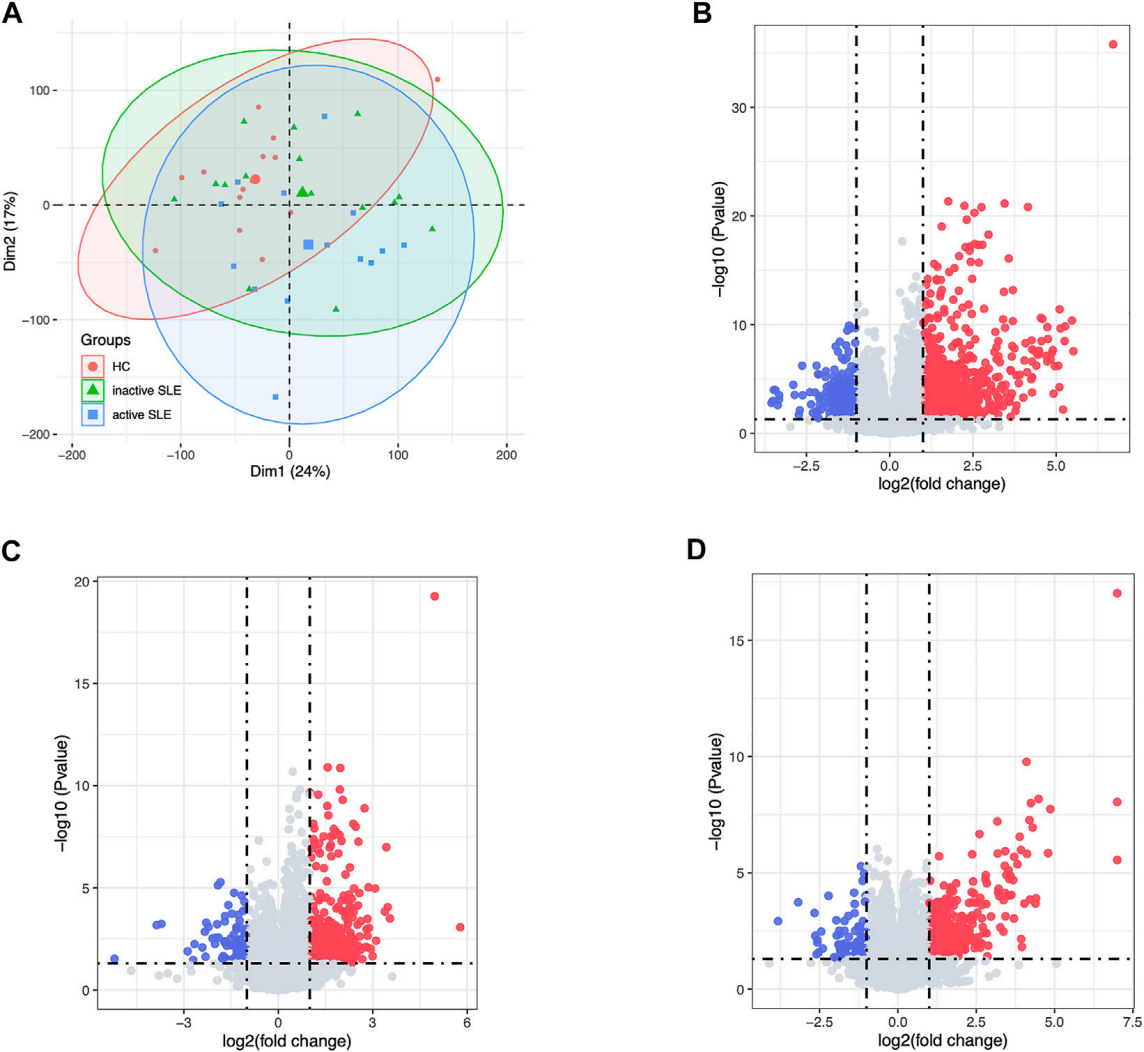
The DEG analysis showed the differences of gene expression profiles in HC, inactive and active SLE patients. **(A)** The PCA result of the gene expression of CD4^+^ T cells in HC, inactive and active SLE patients. **(B–D)** The volcano plot of DEGs between HC and active SLE **(B)**, HC and inactive SLE **(C)**, and inactive SLE and active SLE **(D)**.

Herein, 44 isolated CD4^+^ T cells samples obtained from the dataset GSE97263 were processed. A total of 41,092 Ensemble IDs were converted into gene symbols. As the genes with average raw reads less than 1 were removed, 16,623 genes were selected for the following analysis. After data processing, we examined the gene expression differences in CD4^+^ T cell isolated from HC and SLE patients with different disease activity (Figure 1). According to the PCA results, the gene signatures of these three groups could not be clearly separated (Figure 2A). Next, we analyzed the differences between HC and active SLE group, and found 834 upregulated genes and 252 downregulated genes, with the cut-off criteria of *p* < 0.05 and |log_2_FC| > 1 (Figure 2B). Similarly, the volcano plots showed the difference of DEGs between HC and inactive SLE (Figure 2C), and inactive and active SLE group (Figure 2D). Next, the genes with the largest variance of gene expression were selected for heatmapping, as shown in the Supplementary Figure S1, indicating dynamic changes in gene expression during the development of SLE.

### Identification of hub modules through WGCNA

With no outlier samples, we calculated the optimal soft threshold power (β value) was 8, which was verified by scale-free topology analysis with *R* ^2^ = 0.85 (Supplementary Figures S2A,B). After merging similar modules with the cut-off value = 0.25, 14 modules from the weighted co-expression network were identified based on all the 16,623 genes (Supplementary Figures S2C,D). The gene numbers and detailed symbols in each module were shown in Supplementary Figure S2E and Supplementary Table S1, respectively. In order to explore the relationships among the above-mentioned modules, we quantified the module similarity by eigengene correlation, and the TOM heatmap showed strong correlation within the module groups (Supplementary Figures S2F).

### Correlation between modules of interest and clinical traits

**FIGURE 3:**
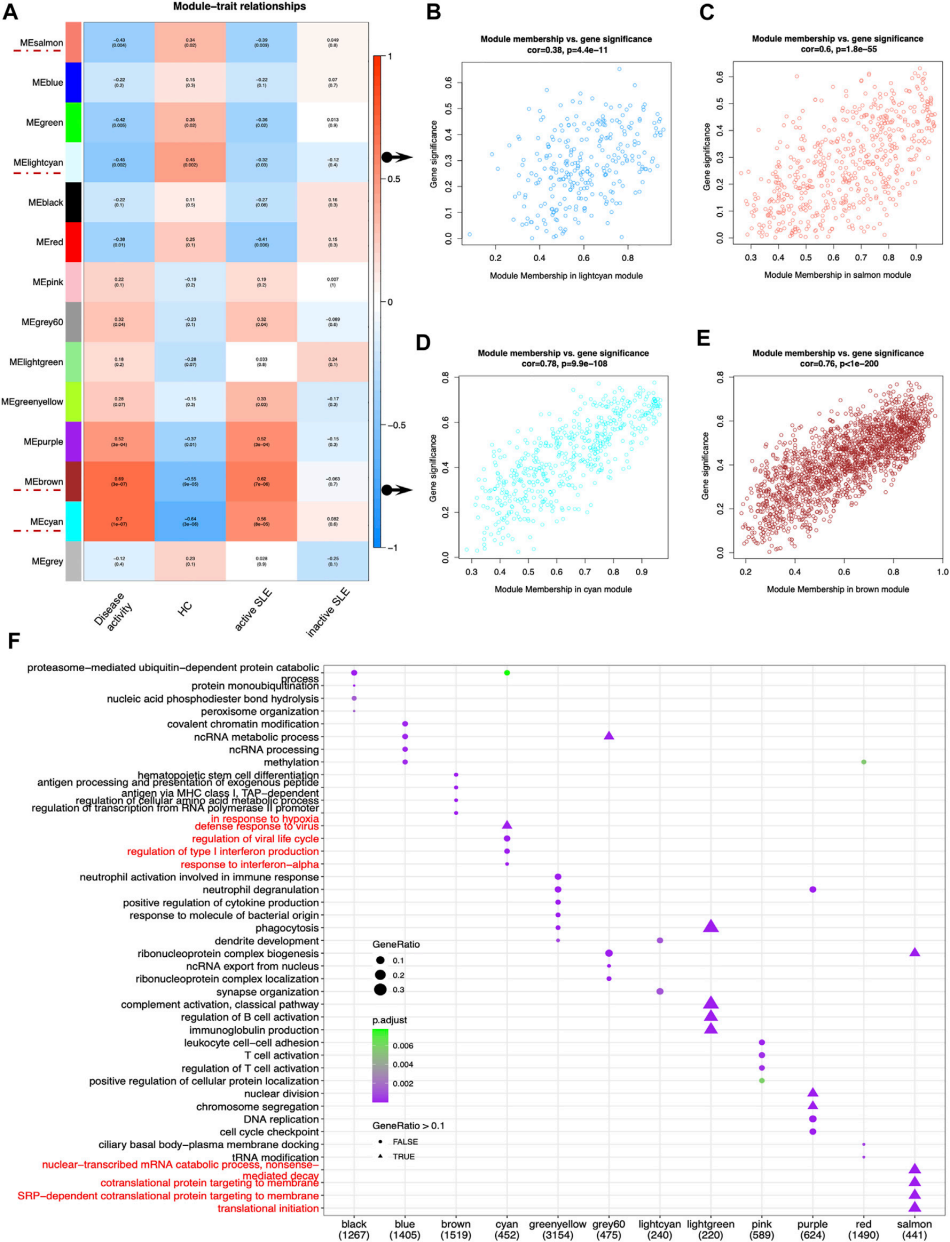
Main findings in the module-trait correlations through WGCNA. **(A)** Module-trait associations. Each row corresponded to a module eigengene (ME), while each column corresponded to a trait. Each cell contained the corresponding correlation and *p* value. The cells were color-coded by correlation according to the color legend. **(B–E)** The scatterplots of Gene Significance (GS) for disease activity *vs*. Module Membership (MM) in the lightcyan **(B)**, salmon **(C)**, cyan **(D)** and brown module **(E)**, which represented significant correlations between GS and MM in these modules. **(F)** The dot plot of GO enrichment analysis of the genes in different modules.

Next, identifying modules most associated with the disease activity is of great biological significance for biomarker development. According to the module-trait relationships in Figure 3A, the MElightcyan and MEsalmon modules were negatively related to disease activity (Cor = −0.45, *p* = 0.002 for MElightcyan, and Cor = −0.43, *p* = 0.004 for MEsalmon), while the MEcyan and MEbrown modules displayed positive relationship with disease activity (Cor = 0.7, *p* = 1*10^–7^ for MEcyan, and Cor = 0.69, *p* = 3*10^–4^ for MEbrown). Thus, these modules were selected for downstream analysis. As shown in Figures 3B–E, GS and MM were highly correlated, illustrating that genes significantly associated with disease activity were also the central elements of modules highly associated with this trait.

### Functional analysis of hub modules

To investigate the correlated biological processes, GO enrichment analysis was carried out on all matched genes in these modules (Supplementary Table S2). In the salmon module, the enriched biological processes were mainly associated with nuclear-transcribed mRNA catabolic process (nonsense mediated decay), cotranslational protein targeting to membrane, SRP-dependent cotranslational protein targeting to membrane and translational initiation. In the cyan module, the biological processes were mainly enriched in the regulation of type I IFN production and response to IFN-α (Figure 3F).

### Identification of gene sets related to SLE disease activity

**FIGURE 4:**
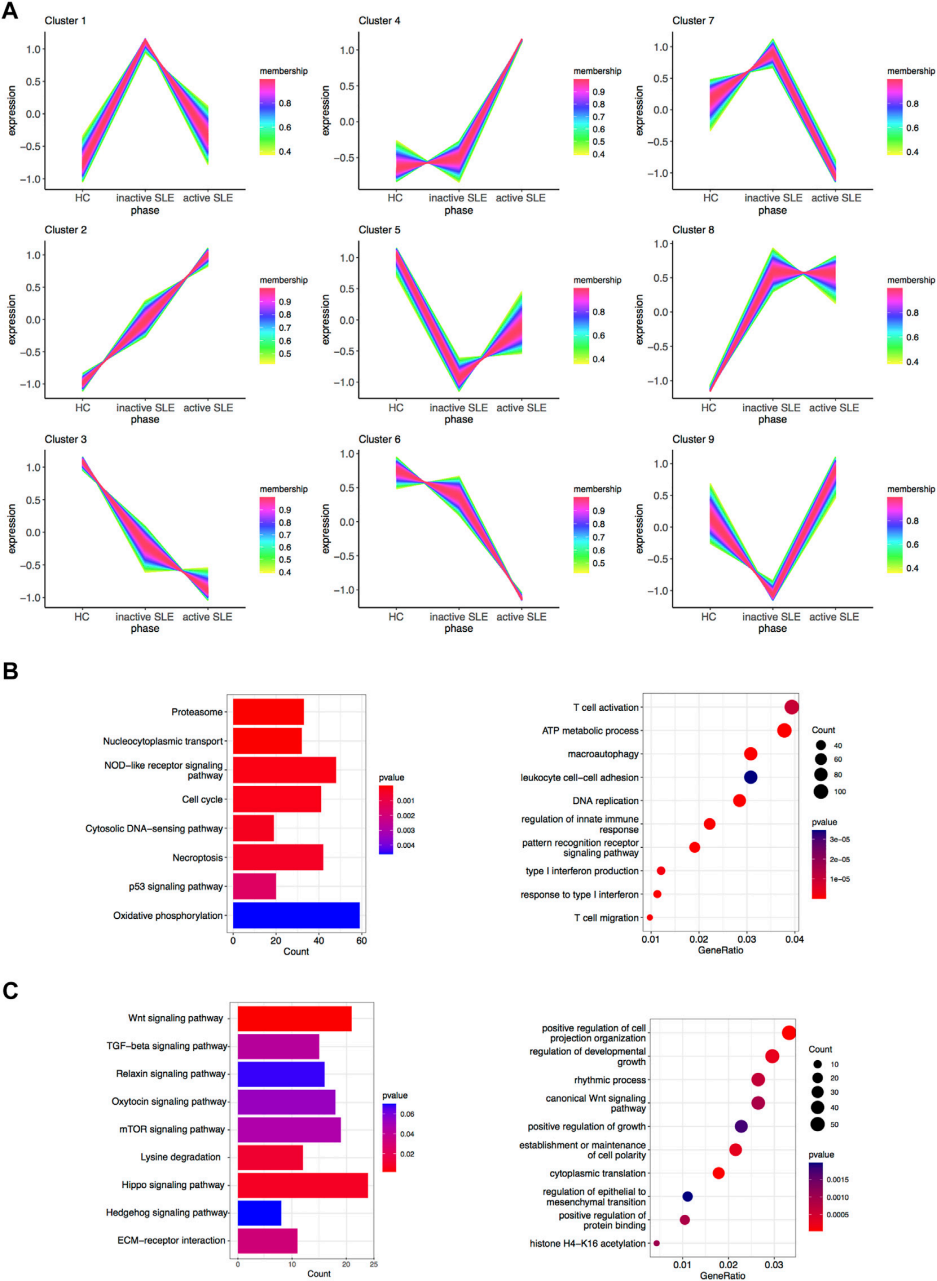
Identification of gene sets related to SLE disease activity. **(A)** Clustering of the gene expression patterns in CD4^+^ T cells from HC, inactive and active SLE patients by TCseq analysis. A total of 9 clusters were obtained and the Cluster 2 and 3 were found significantly correlated with SLE disease activity. **(B)** KEGG (left) and GO (right) analysis of genes in the Cluster 2. **(C)** KEGG (left) and GO (right) analysis of genes in the Cluster 3.

The above WGCNA results showed that the gene expression patterns of hub modules were significantly correlated with disease activity. To further clarify the gene sets closely related to SLE, TCseq analysis was used to analyze CD4^+^ T cells from SLE patients with different disease activity. The detailed gene symbols in different clusters were listed in Supplementary Table S3. The results showed that the Cluster2 was positively correlated with SLE disease activity, while the Cluster3 was negatively related to the disease activity (Figure 4A). In KEGG enrichment analysis, we found that genes in the Cluster2, which were upregulated in SLE patients, were enriched in the pathways of cell cycle, necroptosis and p53 signaling. The GO analysis showed that these genes were enriched in T cell activation, DNA replication, regulation of innate immune response, type I IFN production, response to type I IFN and T cell migration (Figure 4B). In the Cluster3, the KEGG analysis showed that the downregulated genes were enriched in multiple signaling pathways, including Wnt and transforming growth factor-beta (TGF-β) signaling pathways. The GO analysis also revealed that these genes were related to positive regulation of cell projection organization, canonical Wnt signaling pathway, positive regulation of growth and positive regulation of protein binding (Figure 4C).

**FIGURE 5:**
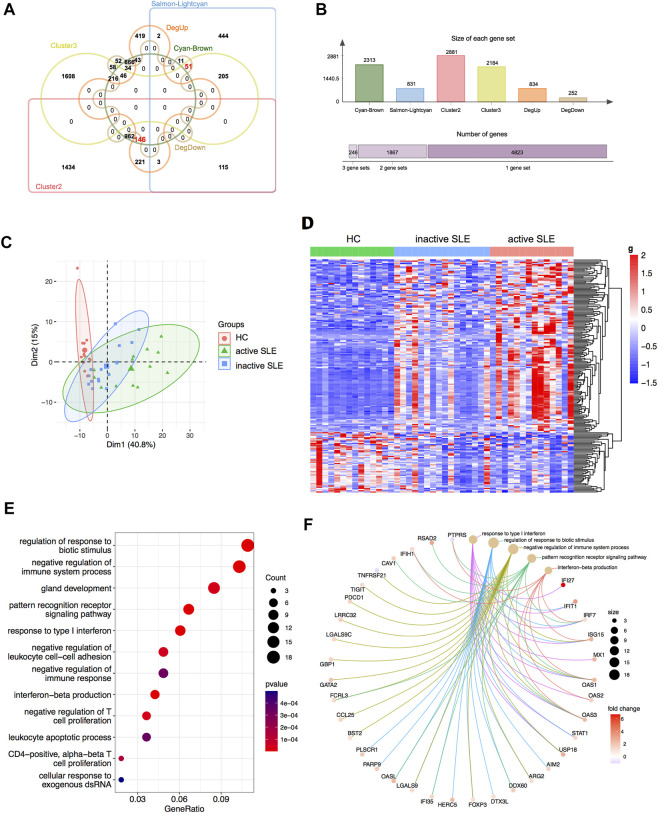
Identification of the candidate key genes. **(A,B)** The venn diagram **(A)** and bar plot **(B)** showed the number of genes in the salmon-lightcyan and cyan-brown modules, Cluster 2 and 3, upregulated and downregulated DEGs gene sets. **(C,D)** The PCA **(C)** and heatmap **(D)** analysis of the selected genes in HC, inactive and active SLE patients. **(E,F)** Dot plot **(E)** and network plot **(F)** of enriched terms of the candidate key genes through GO analysis.

Based on combined analysis of WGCNA, TCseq and DEG, 146 genes showed positive correlation with disease activity and 51 genes showed negative correlation with disease activity in patients (Figures 5A,B). Therefore, these 197 genes could be used to distinguish active and inactive SLE patients (Figures 5C,D). We furtherly analyzed the biological functions of these identified genes and found that they were enriched in the response to type I IFN, IFN-β production, negative regulation of T cell proliferation and immune system process (Figures 5E,F).

### Identification of the potential biomarkers for SLE

**FIGURE 6:**
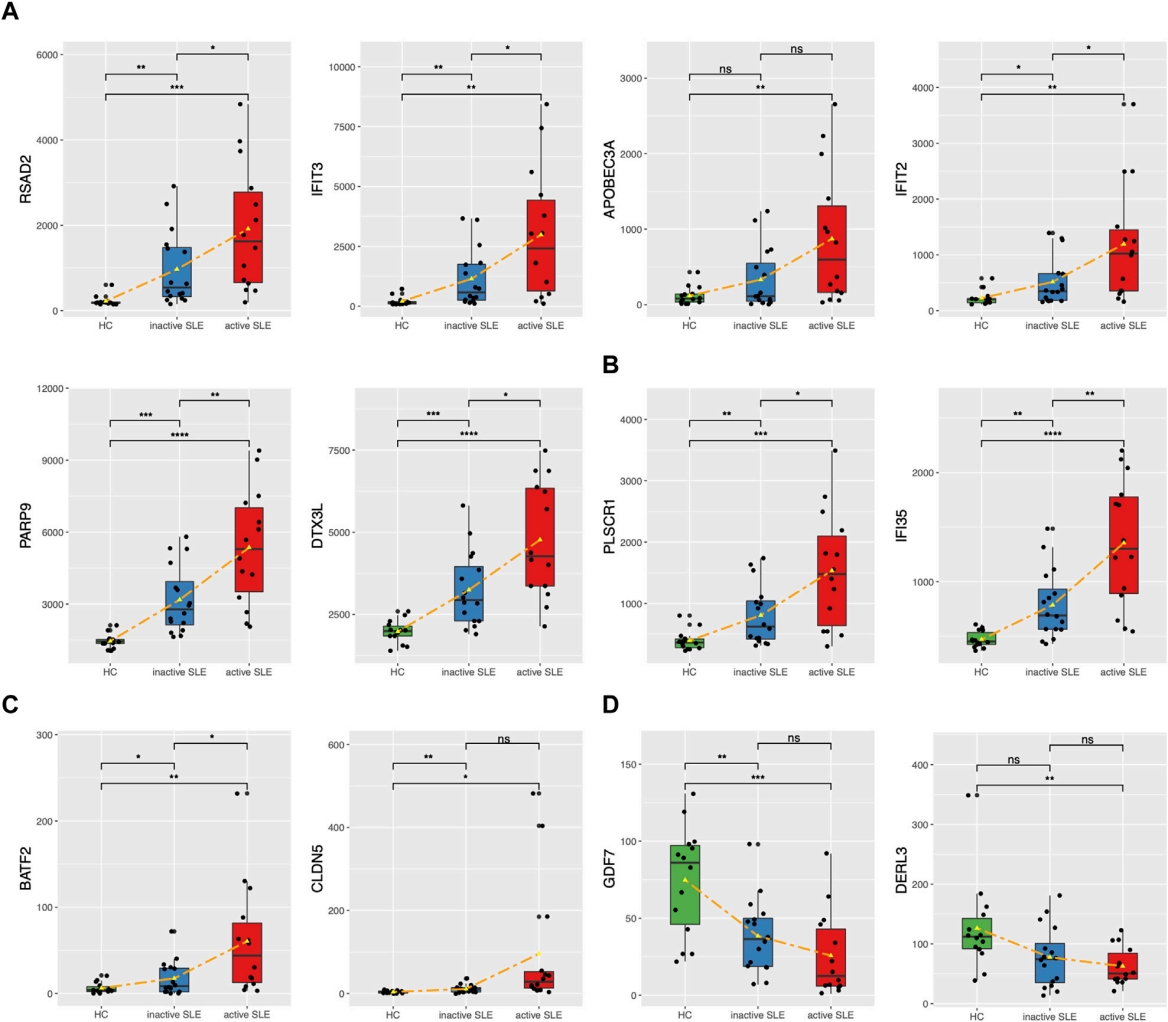
The expression levels of potential key genes in HC and SLE patients. **(A,B)** The expression levels of candidate ISGs in CD4^+^ T cells from HC, inactive and active SLE patients. **(C,D)** The expression levels of upregulated BATF2 and CLDN5 **(C)** and downregulated GDF7 and DERL3 **(D)** in CD4^+^ T cells from HC, inactive and active SLE patients. *p < 0.05; **p < 0.01; ***p < 0.001; ****p < 0.0001.

The genes upregulated in SLE patients mainly focused on type I IFN response. We found 24 ISGs (*RSAD2*, *IFIT3*, *APOBEC3A*, *IFIT2*, *PARP9*, *DTX3L*, *PLSCR1*, *IFI35*, *ISG15*, *CMPK2*, *HERC5*, *GBP1*, *IFI27*, *STAT1*, *MX1*, *IRF7*, *OAS1*, *OAS2*, *OAS3*, *OASL*, *IFIH1*, *SIGLEC1*, *LGALS9* and *BST2*) which were significantly associated with SLE disease activity (Figures 6A,B, Supplementary Figure S3). Among them, *PLSCR1* and *IFI35* were identified as the newfound crucial ISGs. Besides, *BATF2* and *CLDN5* were also identified as key genes (Figure 6C). The expression levels of the above genes were significantly upregulated, especially in those active SLE patients. On the other hand, the expression of *GDF7* and *DERL3* showed significant decrease in active or inactive SLE, compared with that in HC group (Figure 6D). The relevant research in other diseases also pointed that the 4 upregulated genes (*PLSCR1*, *IFI35, BATF2* and *CLDN5*) and 2 downregulated genes (*GDF7* and *DERL3*) exhibited immunoregulatory functions. Therefore, these 6 newfound genes may serve as potential biomarkers of SLE.

## Discussion

It has been reported that lupus CD4^+^ T cells had altered signaling and function, and the hyperactivation of these cells was an important molecular feature of SLE patients. In this study, we analyzed the expression patterns of CD4^+^ T cell in HC, inactive and active SLE through multiple analysis methods, and explored the molecular indicators for potential diagnostic biomarkers and therapeutic targets of SLE.

From methodology aspect, DEG analysis focuses on the differentially expressed genes among different groups, while WGCNA focuses on the correlations between the co-expression modules and the phenotypic and clinical traits, not merely the differences in gene expression profiles. TCseq can be applied for differential analysis between different time points and temporal pattern analysis and visualization of sequencing data. These three bioinformatical tools are complementary to one another to describe key relevant patterns to expanding our capacity for identifying novel biomarkers. In this study, through WGCNA analysis, we established gene expression module-disease activity relationship and found the main functional enrichment in the cyan module included regulation of type I IFN production and the IFN response. This observation is consistent with the current knowledge that ISGs are highly related with SLE disease activity (Crow, 2014; Gkirtzimanaki et al., 2018; Buang et al., 2021). From the perspective of treatment, monoclonal antibodies such as Anifrolumab (Anderson and Furie, 2020; Tanaka and Tummala, 2021) and Sifalimumab (Greth et al., 2017), which block the activation of type I IFNs, have demonstrated significant effectiveness in achieving the composite endpoints in active SLE patients. The medical researchers have also tried to treat lupus by inhibiting type I IFNs in a variety of ways, such as glucocorticoids (Kirou and Gkrouzman, 2013), nicotinamide riboside (Wu et al., 2022) and mTOR inhibitor (Murayama et al., 2020). On the other hand, the salmon module was enriched in biological processes of translational initiation and cotranslational protein targeting to membrane, showing that the expression level negatively correlated with disease activity. The major consequence of perturbing cotranslational targeting for disease progression is so far largely unexploited. We speculate that it may be involved in T cell differentiation, which accompanied by the expression and secretion of a large number of cytokines, as well as communication between cells.

Furtherly, by combining the TCseq with DEG analysis, we identified the presence of 197 genes as closely correlated with disease activity in patients, that may play an integral role in the development of SLE. GO analysis showed that DEGs were mainly involved in negative regulation of immune system process, the response to type I IFN, IFN-β production and negative regulation of T cell proliferation. A total of 24 ISGs showed significant upregulation in SLE patients, especially in those who presented with higher disease activity. The results are consistent with the phenomenon of over-activated IFN response in lupus patients, and further confirm that ISGs play a vital role in the pathogenesis of SLE. Among these ISGs, we identified *PLSCR1* and *IFI35* as the newfound crucial genes. *PLSCR1* is a member of the phospholipid scramblases protein family and involved in regulating phospholipid movements within the plasma membrane. Several reports found that significant hypomethylation of differentially methylated sites in SLE was associated with *PLSCR1*(Yeung et al., 2017; Joseph et al., 2019; He et al., 2022). Besides, elevated expression of *PLSCR1* was found in monocytes from SLE patients (Suzuki et al., 2010), and it was also involved in the modulation of the phagocytic process in differentiated macrophages (Herate et al., 2016). *IFI35* reflects the type I IFN activity induced through the JAK-STAT phosphorylation (De Masi et al., 2021). Elevated expression levels of *IFI35* were found in serum of LN patients, which promoted LPS-caused inflammatory response and cell apoptosis (Zhang et al., 2021). *IFI35* also showed regulatory effects on multiple immune cells by activating macrophages and dendritic cells and promoting naïve T cell differentiation into Th1 and Th17 cells (Xiahou et al., 2017; Jing et al., 2021). A significant elevation in *IFI35* expression in active SLE was also found in our study, indicating that *IFI35* may be associated with the dysregulation of host IFN production and immune cell function in SLE.

Besides, we also explored upregulated *BATF2* and *CLDN5* as critical genes in SLE. *BATF2* was significantly induced and involved in gene regulation of IFN-γ-activated classical macrophages (Roy et al., 2015), and inhibited Th17 responses by suppressing IL-23a expression (Kitada et al., 2017; Kayama et al., 2019). In SLE, *in vitro* experiments indicated *BATF2* may be involved in the impairment of translational and proliferative responses to mitogens in T cells (Ge et al., 2021). *CLDN5* encodes tight junction protein and plays a role in C5a/C5aR1 signaling, which was reported to be related to the impaired brain-blood-barrier (BBB) in SLE with neurological complications (Mahajan et al., 2015). These abnormal expression patterns may eventually disturb immune function with CD4^+^ T cells activation and proliferation. Among these downregulated genes, we identified *GDF7* and *DERL3* as key genes. *GDF7* encodes a secreted ligand of the TGF-β superfamily of proteins. Recent research showed that *GDF7* can exhibit positive regulatory effects on Tregs *via* increasing the expression of *FOXP3* and *CTLA4* (Ding et al., 2021). Thus, the downregulation of *GDF7* in CD4^+^ T cells may lead to impaired suppressive functions of lupus Tregs. *DERL3* encodes proteins belong to the derlin family, which resides in the endoplasmic reticulum (ER). Recent reports using WGCNA or RNA-seq suggested that the function of DERL3 may correlate with plasma cells (Gao et al., 2022; da Silva et al., 2020). Since CD4^+^ T cells act as main helper cells for plasma cell production and cytokines secretion, we suggest that the decreased expression of *DERL3* in lupus CD4^+^ T cells may contribute to the pathogenesis of SLE. Taken together, our data indicated the potential diagnostic and therapeutic value of *GDF7* and *DERL3* in SLE.

There are still several limitations in this study. First of all, the diagnostic or prognostic value of these key genes require a large number of blood samples for validation. Secondly, we did not further clarify the molecular mechanisms of the identified genes. Finally, although we have performed a detailed bioinformatics analysis, some vital genes in the pathogenesis and progression of SLE may still be missed. Thus, further analysis and detailed experiments are needed to definitely establish the predictive biomarkers and explicitly evaluate the performance.

In summary, based on integrated bioinformatical analysis, we found gene sets highly related to SLE disease activity. Besides, some crucial genes mediating the development of SLE were identified, including some previously reported ISGs. In particular, we found 4 upregulated genes (*PLSCR1*, *IFI35*, *BATF2* and *CLDN5*) and downregulated expression of *GDF7* and *DERL3* in SLE patients. Therefore, our findings identified 6 novel potential biomarkers in lupus CD4^+^ T cells, which provided new insights into the development and treatment of SLE. The underlying mechanisms of these genes are still need to be further explored.

## Data Availability

Publicly available datasets were analyzed in this study. This data can be found here: https://www.ncbi.nlm.nih.gov/geo/query/acc.cgi?acc=GSE97263.

## Abbreviations

BATF, basic leucine zipper transcription factor; BBB, brain-blood-barrier; DEG, differentially expressed gene; ER, endoplasmic reticulum; GO, Gene Ontology; HC, healthy controls; IFN, interferon; ISG, interferon signature gene; KEGG: Kyoto Encyclopedia of Genes and Genomes; LN: lupus nephritis; ME, module eigengene; NMI, N-myc-interactor; PBMC, peripheral blood mononuclear cell; PCA, principal component analysis; SLE, systemic lupus erythematosus; SLEDAI, systemic lupus erythematosus disease activity index; TCseq, time course sequencing analysis; TOM, topological overlap matrix; WGCNA, weighted gene co-expression network analysis.
