# Supplementary figures and images for: Identifying key genes in CD4+ T cells of systemic lupus erythematosus by integrated bioinformatics analysis
Source: Front Genet. 2022 Aug 15;13:941221. doi: 10.3389/fgene.2022.941221 (PMC9420982; doi:10.3389/fgene.2022.941221)

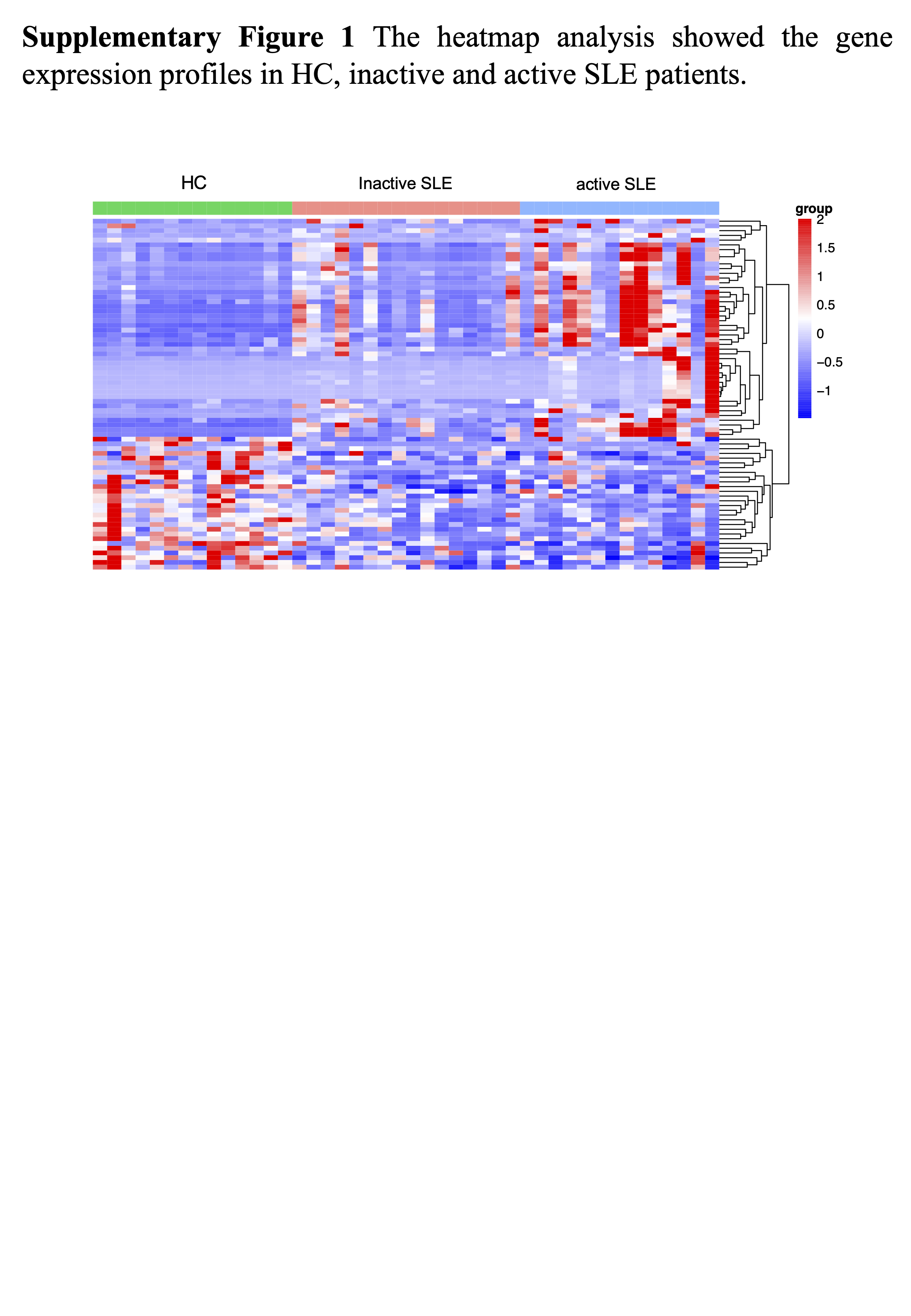

Supplement: Supplementary file 2 [file Image1.TIFF]

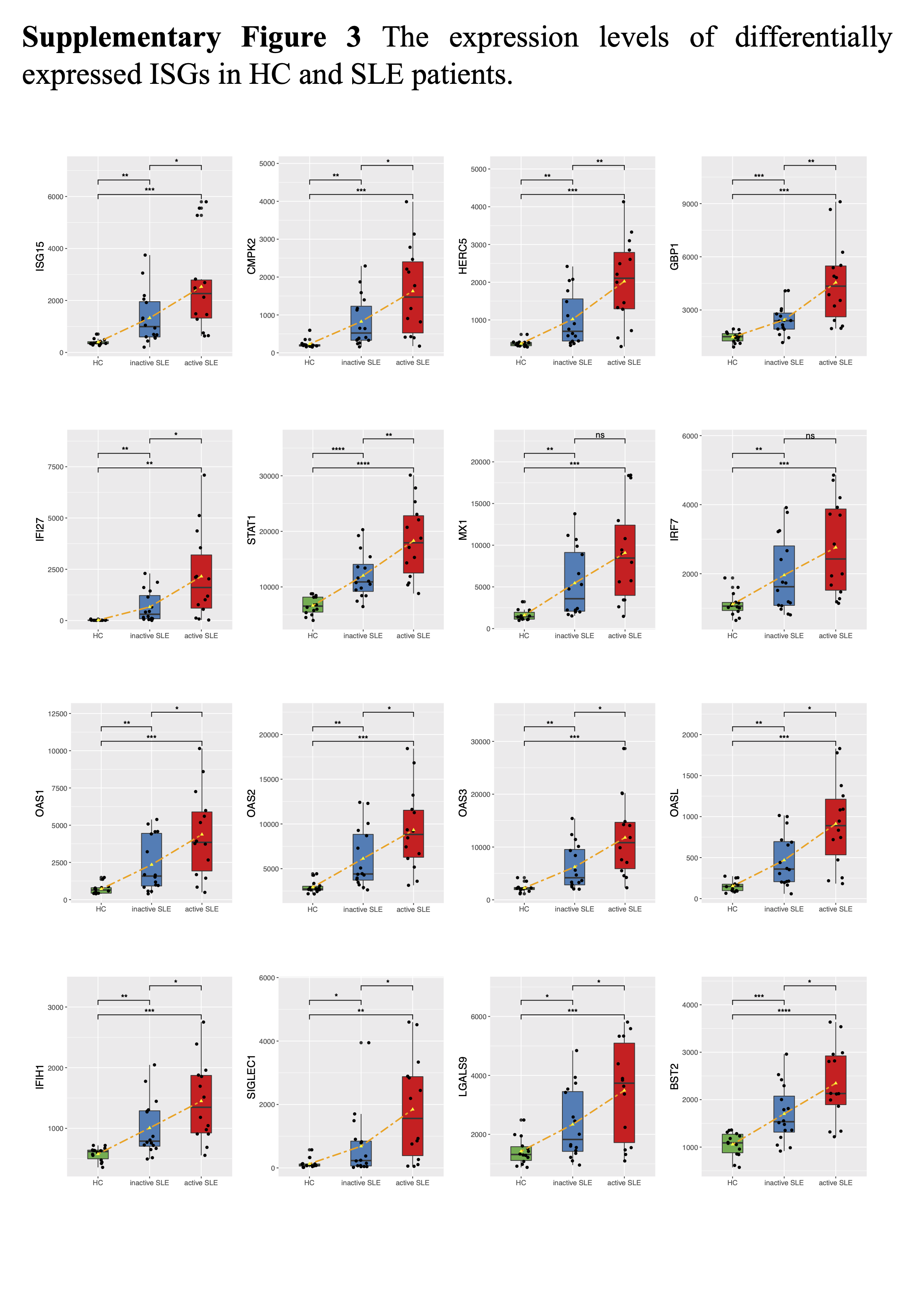

Supplement: Supplementary file 4 [file Image3.tif]

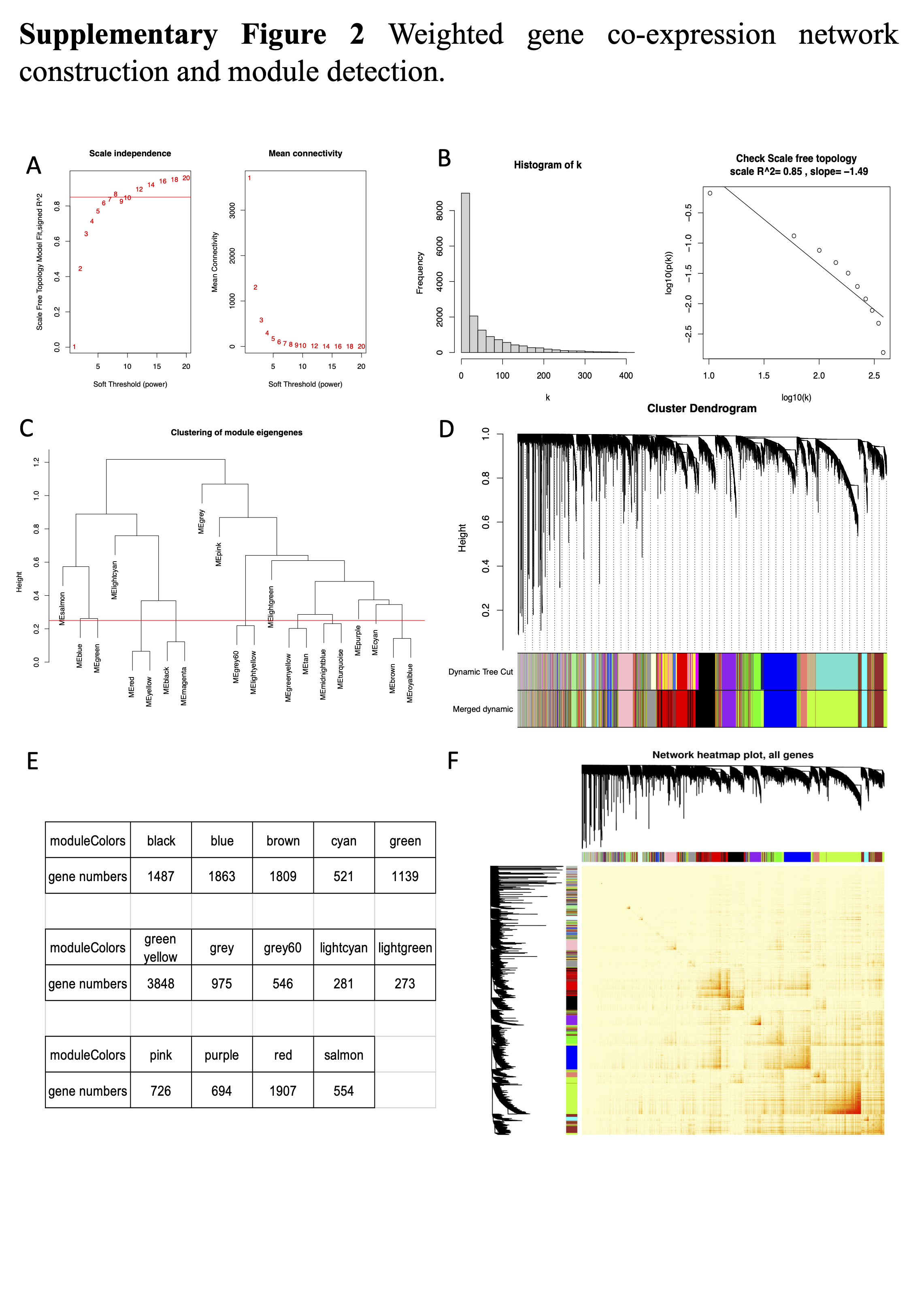

Supplement: Supplementary file 6 [file Image2.TIFF]
