# Supplementary material for: Identifying key genes in CD4+ T cells of systemic lupus erythematosus by integrated bioinformatics analysis
Source: Front Genet. 2022 Aug 15;13:941221. doi: 10.3389/fgene.2022.941221 (PMC9420982; doi:10.3389/fgene.2022.941221)
Supplement: Supplementary file 7 [file DataSheet1.docx]

**Supplementary legends**

**Supplementary Figure S1** The heatmap analysis showed the gene expression profiles in HC, inactive and active SLE patients.

**Supplementary Figure S2** Weighted gene co-expression network construction and module detection.

(A-B) Selection and verification of the most appropriate soft threshold power (β value) to construct a hierarchical clustering. k represented the connectivity of gene network.

(C) Dendrogram of module eigengene (ME) based on dissimilarity measures. The cut-off value was 0.25.

(D-E) Genes were grouped into various modules by hierarchical clustering according to dynamic tree cut (D). The table showed the gene numbers from different modules (E).

(F) Visualizing the gene network using a heatmap plot. The heatmap depicted the TOM among all genes in the analysis. Light colors indicated low overlap and the progressively saturated red colors represented higher overlap among the functional modules. The gene modules were dark-colored blocks that run diagonally. Along the left side and top, the gene dendrogram and module assignment were also displayed.

**Supplementary Figure S3** The expression levels of differentially expressed ISGs in HC and SLE patients.

**Supplementary Table S1** The detailed gene symbols in different modules.

**Supplementary Table S2** GO function analysis annotation of different gene modules.

**Supplementary Table S3** The detailed gene symbols in different clusters analyzed by TCseq.
